# Nasal Sinus Tract of Odontogenic Origin: Report of a Case

**DOI:** 10.1155/2015/813478

**Published:** 2015-11-15

**Authors:** Sagar Sareen, Anjani Kumar Pathak, Parth Purwar, Jaya Dixit, Divya Singhal, Isha Sajjanhar, Kopal Goel, Vaibhav Sheel Gupta

**Affiliations:** ^1^Department of Periodontology, Faculty of Dental Sciences, King George's Medical University, Lucknow, Uttar Pradesh 226003, India; ^2^Department of Conservative Dentistry & Endodontics, Faculty of Dental Sciences, King George's Medical University, Lucknow, Uttar Pradesh 226003, India; ^3^Department of Prosthodontics including Crown and Bridge, Faculty of Dental Sciences, King George's Medical University, Lucknow, Uttar Pradesh 226003, India

## Abstract

Extraoral sinus tract often poses a diagnostic challenge to the clinician owing to its rare occurrence and absence of symptoms. The accurate diagnosis and comprehensive management are inevitable as the aetiology of such lesions is often masked and requires holistic approach. The present case report encompasses the management of an extraoral discharging sinus tract at the base of the right nostril in a chronic smoker. The lesion which was earlier diagnosed to be of nonodontogenic origin persisted even after erratic treatment modalities. Our investigations showed the aetiology of sinus tract to be odontogenic. Initially, a five-step program as recommended by the Agency for Health Care Research and Quality was used for smoking cessation followed by root canal therapy (RCT) and surgical management of the sinus tract. The patient has been under stringent follow-up and no reoccurrence has been noted.

## 1. Introduction

An oral infection may originate in the dental pulp and extend into the periradicular tissues, or it may originate in the superficial periodontal tissues subsequently dispersing through the spongy bone. Thereafter, it may perforate the outer cortical bone and spread in various tissue spaces or discharge onto a free mucous membrane or skin surface [1].

Cutaneous sinus tract of odontogenic origin is rather uncommon and may manifest on adjacent structures like facial skin, maxillary sinus, orbit, and nostril and may also involve distant structures like cavernous sinus that may lead to fatal outcomes [2, 3]. Nasal sinus tract of dental origin may or may not present associated dental pain that often brings about a complex state of diagnostic and management perplexities for the clinicians [4, 5]. Failure to diagnose such lesions can lead to inadvertent treatment. The current case report describes the management of a previously misdiagnosed persistent nasal sinus tract in a chronic smoker patient.

## 2. Case Presentation

**Figure 1:**
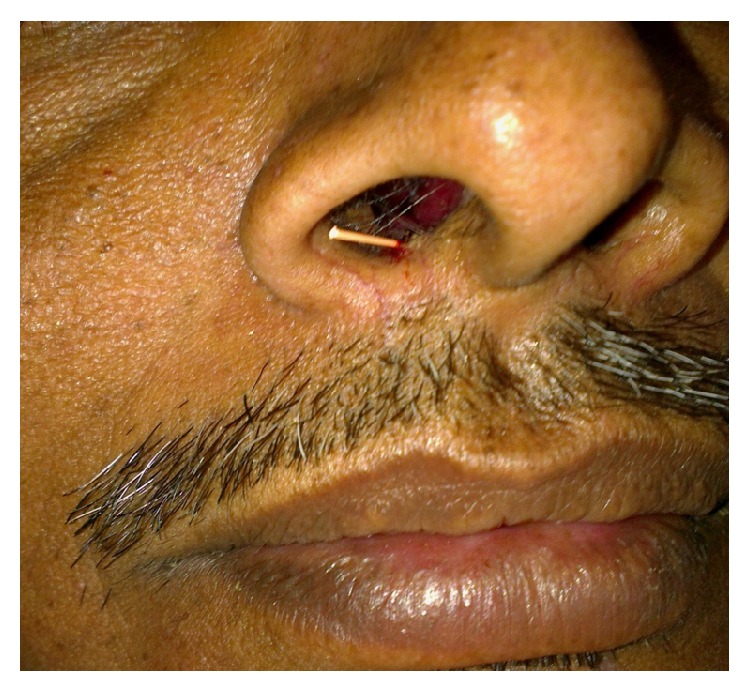
Extraoral view showing sinus tract opening at the base of the right nostril.

**Figure 2:**
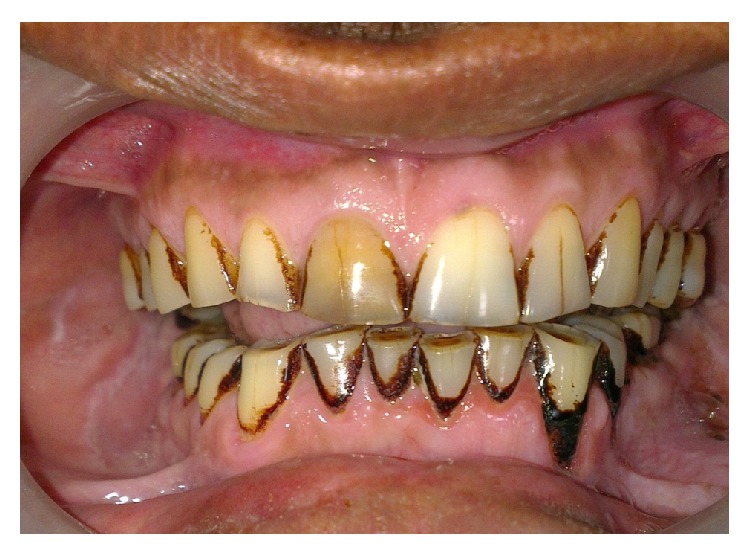
Intraoral photograph showing significantly discoloured maxillary right central incisor.

**Figure 3:**
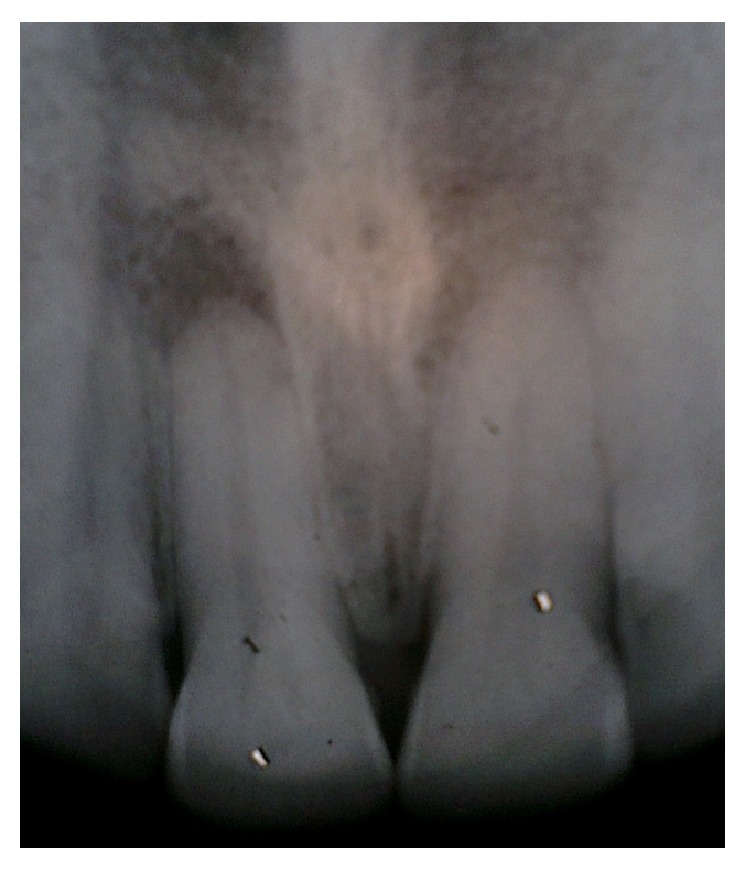
Intraoral periapical radiograph showing the periapical lesion associated with the right maxillary central incisor.

A 40-year-old male belonging to a poor socioeconomic status was referred to the Outpatient Department of Periodontology from ENT Department with a chief complaint of constantly recurring pustule at the base of the right nostril (Figure 1). Further questioning revealed that the pustule used to heal spontaneously within 15 days of eruption and then again exuberated thereafter. A thorough medical history was taken which disclosed that the patient had been under treatment for 6 months from a private practitioner and was prescribed antibiotics as well as intralesional steroids for the constantly recurring pustule which did not yield any positive outcomes. Additionally, the patient was a chronic current and heavy smoker and used to smoke > 20 filtered cigarettes (1 pack) per day for 15 years making it a total of 15 (15 × 1) pack years. Dental history acknowledged that the propositor had a history of trauma to his upper front teeth, when he was in his late 20's, yet no pain, swelling, or any other associated symptom was reported. The propositor had also undergone multiple nugatory biopsies of the concerned lesion only to disclose no abnormality. On extraoral examination, a pustule was detected near the right nostril. Intraoral examination revealed an attrited, noncarious, and significantly discoloured maxillary right central incisor (tooth number 11), bearing prominent enamel cracks running in a coronoapical direction. Generalised attrition with a poor oral hygiene status was also evident by plaque scores and gingival index (Figure 2). On suspecting the patient's nasal sinus tract to be of odontogenic origin, we went in for further investigations. Initially, the pulp vitality tests were performed with the help of heated gutta percha and the results were found to be negative for right maxillary central incisor indicating pulpal necrosis or dental abscess. Intraoral periapical (IOPA) radiograph showed distinct periapical radiolucency with compromised periodontal status in relation to tooth number 11 (Figure 3). The above-mentioned investigations further confirmed the diagnosis of a periapical abscess of odontogenic origin in relation to the right maxillary central incisor escaping through an extraoral nasal sinus tract.

## 3. Differential Diagnosis

Differential diagnosis may include suppurative apical periodontitis, osteomyelitis, pyogenic granuloma, congenital fistula, salivary gland fistula, infected cyst, and deep mycotic infection [4, 6]. Foreign-body lesions, basal cell carcinoma, squamous cell carcinoma, and granulomatous disorders may appear similar to a draining sinus tract of dental origin but are not true sinus tracts [7]. A draining sinus in the head and neck region warrants a suspicion of tract being of odontogenic origin arising due to pulpal necrosis.

## 4. Treatment, Outcome, and Follow-Up

**Figure 4:**
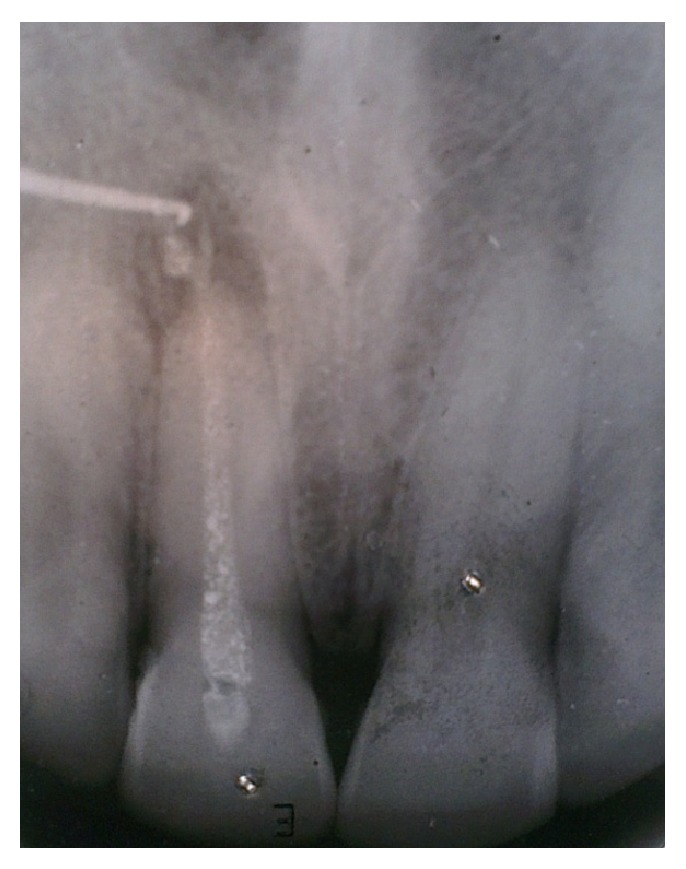
Radiograph showing gutta percha tracing of the sinus tract.

Initially, oral hygiene instructions were given followed by scaling and root planing (SRP) to ensure a plaque free operating site. The patient was recalled to check the response of periodontal tissues which were found to be in healthy condition. The patient was recalled after 1 week for initiation of endodontic therapy. He was actively enrolled in smoking cessation program. Conventional root canal therapy was initiated with the preparation of a palatal access cavity. The canal was thoroughly irrigated with physiologic saline and 2% chlorhexidine solution. During third recall visit, the canal was reirrigated and biomechanical preparation of the single canal was completed. An intracanal medicament consisting of a mixture of nonsetting calcium hydroxide, glycerine, and iodoform was given and access cavity was sealed temporarily with zinc oxide eugenol cement (Figure 4). During the same visit, the nasal sinus tract was traced with a gutta percha point to reconfirm the diagnosis pertaining to its constant persistence (Figures 1 and 4). The patient was then recalled after 7 days for further evaluation, which showed that the nasal sinus discharge still persisted. The intracanal dressing was changed once weekly for 4 weeks consecutively, only to find a persistent nonhealing sinus. The unsatisfactory results lead us to shift our treatment plan towards surgical endodontic therapy. The tooth was obturated till the predetermined working length and a periradicular debridement procedure was planned; a full thickness mucoperiosteal trapezoidal flap was elevated which disclosed granulation tissue filled periapical cavity with absence of the associated buccal cortical plate. The defect was debrided completely with the help of curettes. Apical 3 mm of the root was resected which had circumscribing endoperio lesion. The flap was then repositioned, compressed, and sutured at presurgical position. Routine postoperative instructions were given and appropriate medications were prescribed which included amoxicillin 500 mg, metronidazole 400 mg thrice daily, and a combination of diclofenac and paracetamol twice daily for a period of 3 days.

**Figure 5:**
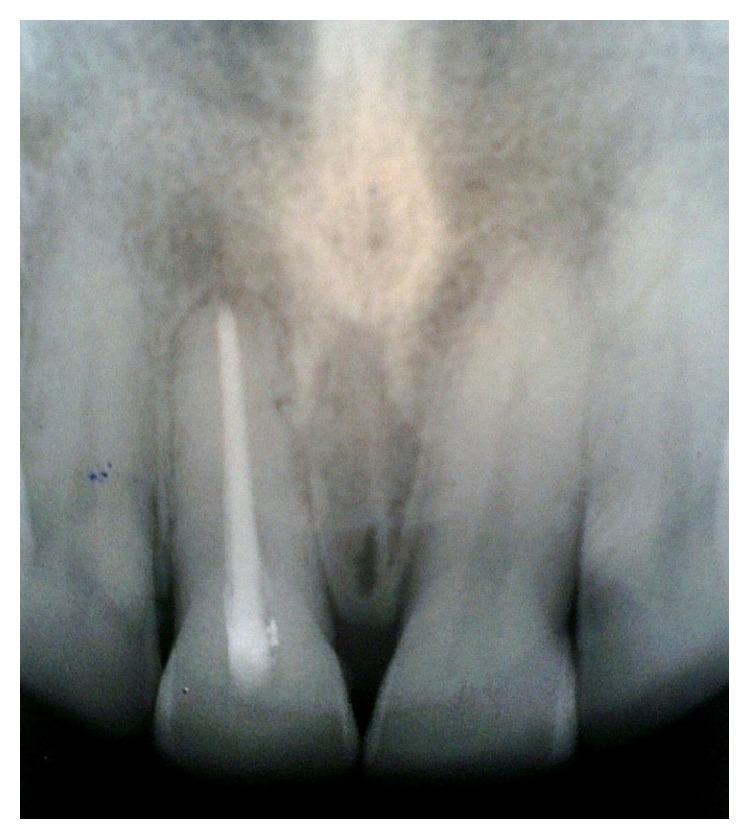
Postoperative radiograph revealing complete healing of the defect at 1-year recall visit.

**Figure 6:**
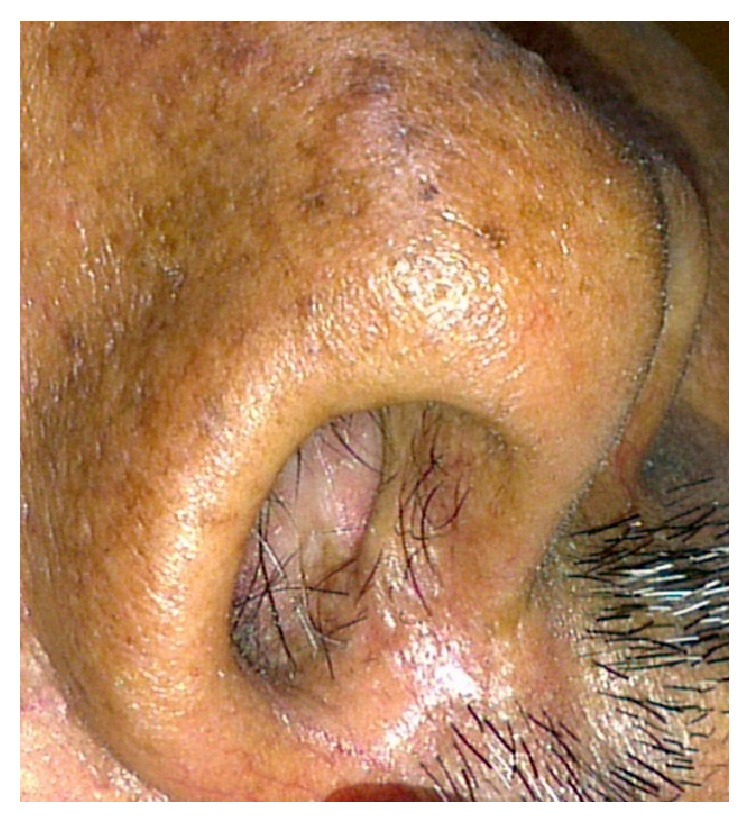
Healed nasal sinus tract with no signs of recurrence at 1 year.

The sutures were removed 1 week postoperatively. Healing took place uneventfully with minimal discomfort to the patient and the nasal sinus opening ameliorated satisfactorily. In later appointments, the tooth was restored with aesthetic restorative material. Clinically and radiographically, the lesion had healed entirely at 1-year recall visit and no recurrence had been noted along with achievement of complete cessation of smoking (Figures 5 and 6). Additionally, the propositor actively participated in the 5 As' program and was able to completely stop smoking 5 months after its implementation.

## 5. Discussion

The patient herein presented with a discoloured maxillary right central incisor without pain, which became a diagnostic dilemma. He was unable to relate the nasal lesion with the teeth due to the absence of any dental symptoms. Drainage of periapical infection by perforation of bony plates occurs along lines of least resistance. The attachment of muscles may determine the route that an infection will take, channelling it either intraorally or extraorally [1]. In the present case, a trauma induced periapical infection of maxillary central incisor manifested as a nasal sinus tract owing to the anatomic proximity between the tooth root and the nasal floor. The gutta percha tracing of the tract confirmed its odontogenic origin.

The most common cause of sinus tract of odontogenic origin was found to be chronic periapical abscess as reported in 71% of the cases as evaluated by Slutzky-Goldberg et al. [8]. Secondary causes of pulpal necrosis included trauma and periodontal infections [9]. Sinus tracts originate more commonly from infected mandibular teeth than maxillary teeth [10]. The present report had a maxillary anterior tooth involved thereby making it uncommon and a must know variability of the periapical infections for the clinicians.

Due to the previously made incorrect diagnosis, the propositor had undergone erratic treatment course which included multiple biopsies and long-term antibiotic therapy. With such misdiagnosis, the patient may undergo unnecessary treatment like radiation therapy, electrodesiccation, intralesional injection of steroid, and oral steroid [2, 3, 11]. Misdiagnosis and wrong treatment due to absence of dental symptoms and unusual presentation are an unfortunate but highly prevalent occurrence [11].

The dangerous area of the face consists of the area from the corners of the mouth to the bridge of the nose, including the nose and maxilla [12]. Due to the special nature of the blood supply to the human nose and surrounding area, it is possible for retrograde infections from the nasal area to spread to the brain causing cavernous sinus thrombosis, meningitis, or brain abscess. The anatomic location of the sinus placed the propositor under high risk of life threatening conditions. Furthermore, the overall systemic health of the patient was also compromised due to negative effects of heavy smoking on the immune status of the host. The program as recommended by the Agency for Health Care Research [13] was used as an approach for cessation of smoking. This program uses the five As approach for smoking cessation: (1) ask (to identify the propositor's tobacco use status), (2) advise (on associations between smoking and negative impact on systemic health along with the benefits of smoking cessation), (3) assess (propositor interest and willingness to participate in smoking cessation programs), (4) assist (use of appropriate techniques to assist patient in tobacco cessation), and (5) arrange (follow-up contacts with the patient).

Nonsurgical endodontic therapy complemented by surgery or dental extraction is the treatment of choice for extraoral sinus tracts [6]. Spontaneous closure of the tract should be expected within five to fourteen days after root canal therapy or extraction [14]. The tooth in the present report had to be resorted to surgical means considering the persistence of lesion for more than 4 weeks even after the root canal treatment which forms an indication for apical surgery as revised by European Society of Endodontists in 2006 [15].

The limitations of the present case could be the use of 2% chlorhexidine solution as a root canal irrigant.

Ideal requisites for an endodontic irrigant are that it (a) should possess antimicrobial activity, (b) should have ability to dissolve necrotic and vital pulp tissues, (c) should remove smear layer, (d) should be nontoxic and biocompatible, and (e) should serve as a lubricant [16]. Chlorhexidine lacks a tissue dissolving property [17] and does not remove the smear layer [16]. The use of other irrigating solutions such as sodium hypochlorite at concentrations 1%–5.2% may be considered more efficient.

A nasal sinus tract of odontogenic origin is an extremely rare presentation of a common disease of periradicular tissues as presented elsewhere [18, 19] and warrants careful diagnosis and management. Hence, we conclude that apical surgery is a predictable treatment option for teeth with apical pathology that cannot be managed by conventional, nonsurgical endodontics.
